# Ghost messages: cell death signals spread

**DOI:** 10.1186/s12964-022-01004-0

**Published:** 2023-01-09

**Authors:** Mingming Zhang, Yuan Lin, Ruijing Chen, Haikuan Yu, Yi Li, Ming Chen, Ce Dou, Pengbin Yin, Licheng Zhang, Peifu Tang

**Affiliations:** 1 Department of Orthopedics, Chinese PLA General Hospital, Beijing, 100853 People’s Republic of China; 2 National Clinical Research Center for Orthopedics, Sports Medicine and Rehabilitation, Beijing, 100853 People’s Republic of China; 3 Department of Orthopedics, The Second Affiliated Hospital of Harbin Medical University, Harbin, 150001 Heilongjiang People’s Republic of China; 4 Department of Orthopedics, Southwest Hospital, Army Medical University, Chongqing, 400038 People’s Republic of China

**Keywords:** Cell death, Ghost message, Intercellular communication, Extracellular vesicle, Soluble factor

## Abstract

Cell death is a mystery in various forms. Whichever type of cell death, this is always accompanied by active or passive molecules release. The recent years marked the renaissance of the study of these molecules showing they can signal to and communicate with recipient cells and regulate physio- or pathological events. This review summarizes the defined forms of messages cells could spread while dying, the effects of these signals on the target tissue/cells, and how these types of communications regulate physio- or pathological processes. By doing so, this review hopes to identify major unresolved questions in the field, formulate new hypothesis worthy of further investigation, and when possible, provide references for the search of novel diagnostic/therapeutics agents.

**Video abstract**

**Supplementary Information:**

The online version contains supplementary material available at 10.1186/s12964-022-01004-0.

## Introduction

**Fig. 1:**
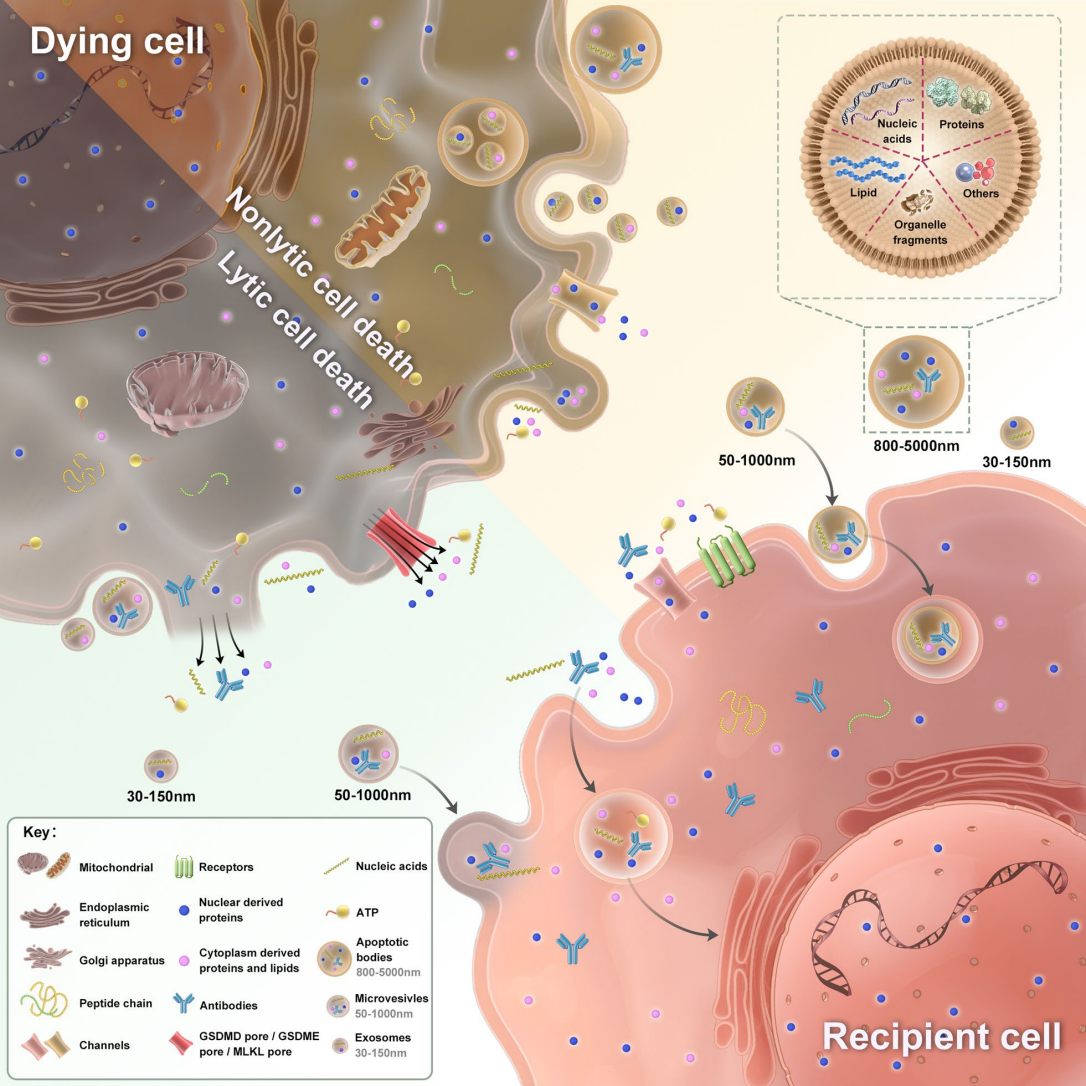
Schematic of ghost messages from dying cells modulating the function of recipient cells

Ghost messages, information from dying or dead cells, will be sent to viable and healthy cells in local and remote microenvironment to affect their biological functions [1]. To maintain body homeostasis, cells are in constant communication and interaction with each other through signaling molecules, which include cell death signals. Cell death is indispensable for growth and development, as well as fighting against various internal and external stimuli [2, 3]. A myriad of cell death pathways have been reported so far, and despite the strikingly different signaling cascades involved, they all share a common feature, the release of bioactive molecules [4]. Hence the question; what kind of information do these molecules convey, which receptors do they bind to, and which cells do they target? Accumulating evidence suggests that ghost messages engage in multiple biological responses and are involved in the occurrence and development of diseases [5, 6]. In this review, we classify the cell death based on their common features; summarize the ghost messages focusing on extracellular vesicles and soluble factors; and discuss the relationship between this kind of intercellular communication and diseases (Fig. 1).

## Features of cell death

Cell death is traditionally divided into two types, regulated cell death which is a genetically regulated process with the finely controlled signaling pathways, and accidental cell death typically causing a strong immune response, whereas regulated cell death can also activate an inflammatory response under specific circumstances, which is usually referred to as immunogenic cell death [7, 8]. Although molecular mechanisms of cell death vary from each other, they ultimately converge on a lytic or nonlytic morphological feature [9, 10]. Based on their end-point features, whether the plasma membrane breakdowns and the cellular contents are released, cell death has been divided into lytic cell death or nonlytic cell death [10–12].

Different forms of lytic cell death have been reported, including necrosis, secondary necrosis, necroptosis, ferroptosis and pyroptosis. The cellular and molecular mechanisms of each of these processes have been elegantly defined by different research groups, but they all share a common feature, that the dying cells ultimately lose membrane integrity and release damage-associated molecular patterns (DAMPs), proinflammatory cytokines, autoantigens and other cellular contents into the extracellular microenvironment [13]. Some of such molecules are the high mobility group box 1 protein (HMGB1), adenosine triphosphate (ATP), purine metabolites, heat-shock proteins (HSP) and interleukin-1β (IL-1β). In response to internal or external stimuli, cells activate the cell death pathway signaling cascades modifying the extracellular microenvironment, recruiting resident and circulating immune cells and triggering tissue damage and repair via these molecules. Under pathophysiological conditions, this process releases distinct molecules that can lead to autoimmune diseases [3, 14].

Nonlytic cell death such as apoptosis and autophagy, maintains membrane integrity and does not elicit vigorous inflammatory responses [15]. These two cell death programs are highly coordinated by distinct signal molecules and characterized by formation of apoptotic bodies (ApoBDs) and autophagosomes which are involved in transport, degradation and secretion of biomolecules [16, 17]. Apoptosis, a programmed cell death which is mediated by the intrinsic and extrinsic signaling pathways, is essential for tissue homeostasis and immune tolerance, accompanied by condensation of the chromatin, nuclear fragmentation, phosphatidylserine (PS) externalization, and ApoBDs formation [18]. Autophagy is a major catabolic pathway responsible for the elimination of long-lived and damaged proteins and organelles by autophagosomes and lysosomes, and also acts as a protective mechanism in response to various stresses including starvation, oxidative stress, hypoxia, or infection [19, 20].

In a multicellular organism, immune cells, endothelial cells, hepatocytes, and tumor cells inevitably undergo cell death in normal cell turnover, tissue homeostasis and disease progression. Accumulating evidence suggests that these dying cells play a regulatory role in inflammation, regeneration, repair and tumorigenesis [21, 22]. Thus, further researches in the concrete mechanisms of regulation are likely to provide novel therapeutic targets and diagnostic markers for the corresponding diseases. In this review, novel intercellular communication signals from dying cells, and diversified biological outcomes in diseases are discussed in detail.

## Forms of ghost messages

Intercellular communication is a well-defined physiological activity, indispensable for multicellular organisms to maintain health and homeostasis [23]. Mechanical, metabolic and biochemical stimuli from individual cells influence their surrounding neighbors, regulate physiological functions and pathological outcomes [24]. Adjacent cells recognize, receive, process and transduce these molecules, thereby initiating downstream biological responses and inducing adaptive changes, such as cellular recruitment and polarization, signaling pathway activation and regulation, and epigenetic modifications [24, 25].

Under physiological and pathophysiological conditions, cell death is inevitable and indispensable, conferring advantages or disadvantages to organisms. Whichever kind of cell death process will always be accompanied by the release of various molecules or extracellular vesicles. Most of these cellular components can signal directly or carry bioactive molecules to signal indirectly. After binding to membrane receptors of neighboring cells, or being engulfed by recipient cells, these molecules influence downstream functions through intercellular communications and signal transduction [26]. In general, intercellular interactions between dead cells and their healthy neighbors are mediated by extracellular vesicles and soluble factors released actively or passively [27]. However, other contact-dependent communications, such as gap junctions, tunneling nanotubes and cell–cell adhesion between dead cells and viable cells, have not yet been reported.

### Extracellular vesicles

Extracellular vesicles (EVs) are cell-derived lipid bilayer-enclosed membranous structures that consist of ApoBDs, exosomes and microvesicles carrying biomolecules such as nucleic acids, proteins and lipids [28]. Originally viewed as mere “garbage bags”, it is now recognized that these vesicles can mediate intercellular communication and horizontal gene transfer whether in natural or engineering form [29, 30]. (Additional file 1: Table S1 and S2**)** Vesicle secretion is a constitutive physiological process of healthy cells, but dying cells also release Evs [31]. Several studies have shown that cells undergoing lytic cell death generate a larger number EVs compared with viable and apoptotic cells [27]. EVs-dependent communication is extremely efficient, as the cargos are protected by a membrane and surface proteins allow for a targeted delivery [32]. Despite uniformly classified, EVs can vary significantly in diameter, contents and functions, depending on types and physiological or pathological state of the parental cell.

#### Apoptotic bodies (ApoBDs)

The late stage of the caspase-dependent apoptotic pathway is marked by ApoBDs enclosing residual components of dead cells, which present another available means of cell–cell communication [33, 34]. Compared with exosomes and microvesicles, ApoBDs have a diameter of 800–5000 nm and are generally assumed to be phagocytosed to prevent adverse impacts on the microenvironment [35]. In addition to bioactive nucleic acids, proteins and lipids, ApoBDs contain some autoantigens such as histone 1, histone 2B and histone 3 [33]. It is still unclear if the sorting process of cellular contents into ApoBDs can be regulated. A recent study shows that Pannexin-1 channel inhibitor trovafloxacin increases the proportion of ApoBDs containing DNA and nuclear proteins, and generates larger number of smaller ApoBDs [36]. Georgia K et al*.* also reported a novel way to generate ApoBDs via a beads-on-a-string structure, which facilitated a sorting process to exclude nuclear contents from ApoBDs [37].

ApoBDs initiate an array of biological processes through functional biomolecules [38], but how do ApoBDs function as signal vehicles? During the process of apoptosis, dying cells actively secret cytokines that act as the “find-me” signal to set up a chemotactic gradient, which recruits engulfing cells to limit local inflammation and clear dead cell debris [39, 40]. In the meantime, apoptotic cells expose PS in membrane surface of ApoBDs acting as an “eat me” signal, which is recognized and engulfed in a process called efferocytosis by professional, non-professional and specialized phagocytes [41], such as macrophages, dendritic cells, endothelial cells, fibroblasts and bone marrow mesenchymal stem cells (MSCs) [40, 42]. PS on ApoBDs interacts with PS receptors such as BAI1, Stabilin-2, αvβ3 Integrin, αvβ5 Integrin, Tim4 and MerTK and other molecules, GULP, FAK, MFG-E8 and Gas6 [43]. For example, BAI1, 7-transmembrane G-protein coupled receptor (GPCR), facilitates actin cytoskeletal rearrangements and corpse internalization through ELMO/Dock180/Rac signaling pathway [44, 45]. Aside from PS, annexin I, calreticulin (CRT) and phophoethanolamine (PE) on the surface of ApoBDs also act as critical “eat-me” signals mediating efferocytosis [46].

Recent bioinformatics analysis showed that parental cells derived ApoBDs exhibit a highly similar transcriptome and function as the parental cells [34]. Meantime, researchers more precisely analyze the transcriptional profiling of ApoBDs from different cells, for example, lncRNAs and mRNAs are the dominant RNA in osteoclast derived vesicles [34]. However, the function of miRNA in ApoBDs has been poorly reported and requires further in vivo and in vitro elucidation. Taken together, ApoBDs seem to mimic their parental cells, carrying bioactive molecules to signal to and communicate with recipient cells.

#### Exosomes and microvesicles

Exosomes and microvesicles are widely found in biofluids, ranging from 30–150 to 50–1000 nm in diameter respectively [47, 48]. They can engage in different biological processes, target nearby cells or perform long-distance communication which attributed to their ability to carry bioactive cargos [30, 49].

Involved in both physiological and pathological processes, exosome secretion is a common phenomenon with a potential impact on tumor therapy, diagnostic biomarkers and regenerative medicine [50, 51]. Unlike ApoBDs, cell death is not required for exosome formation [52], but caspase activation can induce a paracrine apoptotic response, the release of apoptotic exosome-like vesicles, with a size of 30–100 nm [53]. However this apoptotic exosome-like vesicles express distinct protein signatures and lack some markers of classical exosomes [53]. In addition, pyroptotic exosomes, in which immune response related proteins are also enriched, directly activate NF-κB signaling pathway and engage in immune response [54, 55].

Microvesicles, also termed as microparticles, are released from cells upon activation or during apoptosis and autophagy [56, 57]. For example, endothelial cell‑derived microvesicles, released during endothelial cell apoptosis, play a role in the progression of atherosclerosis [58]. Importantly, microvesicles of different origin are distinct, with strikingly different bioactive contents and different functional properties [59]. Apart from apoptosis, other lytic cell death pathways [27], such as pyroptosis, secondary necrosis, ferroptosis and necroptosis, can release microvesicles carrying caspase-1, GSDMD, ASC, mitochondria DNA, ferritin and HMGB1 [56, 60, 61]. Thus, the release of microvesicles is common in the process of cell death, which will engage in signal communication and potentially impact distinct physical responses.

### Soluble factors

A variety of soluble factors secreted by viable cells, such as cytokines, growth factors, receptors, hormones and metabolites can affect specific target cells via autocrine, paracrine, endocrine signals and neurotransmitters [21, 24].

Dying cells transmit messages through active secretion of some factors or passive release of intracellular contents following loss of membrane integrity [39, 62]. During the cell death process, cells consume ATP to drive transcription of bioactive molecules, including pro- and anti-inflammatory factors [63]. For example, extracellular stimuli activate the NF-κB pathway initiating the transcription of pro-caspase-1 and pro-IL-1β in the canonical pyroptosis pathway [64]. During apoptosis, apoptotic cells recruit phagocytes that remove damaged cells and initiate remodeling through releasing a myriad of “find-me” signals, such as nucleoside triphosphates (ATP and UTP), lysophosphatidylchloline (LPC), fractalkine and the chemokine CX3CL1 [39, 65]. Pannexin-1channels, under the control of effector caspase-3 and -7 during apoptosis, open to release cellular ATP [39, 66]. Meanwhile, ATP degradation products, including ADP, AMP and adenosine, serve as an anti-inflammatory metabolites [67]. Similarly, other metabolites released by apoptotic cells, like the polyamine pathway end-product spermidine, also plays an anti-inflammatory role [68]. Medina et al*.* profiled the apoptotic metabolite secretome and found several conserved metabolites, including AMP, GMP, creatine, spermidine, glycerol 3-phosphate and ATP, which modulated multiple gene programs in adjacent healthy cells, such as inflammation and tissue repair [69]. Interestingly, Anderson et al*.* showed that in dying mammalian cells, these metabolites acted as nutrients for bacterial growth [70].

Autophagic vacuole contents are secreted via exocytic process, such as Acb1, IL-1β and α-synuclein proteins, which is a form of unconventional secretion utilizing autophagic machines [71, 72]. Other forms of cell death tend to show pro-inflammatory features through DAMPs secretion [73]; e.g. (1) secreting cytokines during the early progress; (2) releasing HMGB1, ATP, UTP, LDH, AST, ALT, HSPs, caspase-1, IL-1β, IL-33, type I IFNs and NLRP3 inflammasome proteins; (3) exposing potentially pathological neoantigens/neoepitopes such as actin filaments; (4) shedding from the affected cell surface, such as soluble membrane receptors into the extracellular space [74–76]. However, DAMPs are also released by damaged and senescent cells, therefore, whether some soluble factors are released solely upon cell death deserves further investigation.

The general mechanisms of viable cells secreted signals acting on downstream receptors have been clearly elucidated, such as GPCRs, ion channels and specific transporters[77]. However, signaling molecules released by dying cells actively engaging in cellular interactions is less well known and attracting wide attention. Some of these soluble factors also act on the same receptors, such as P2Y-GPCRs and P2X ion channels [66]. Moreover, dying cells derived DAMPs are able to bind to pattern recognition receptors (PRRs), such as Toll-like receptor (TLR), Nod-like receptor (NLR), absent in melanoma 2 (AIM2) and C-type lectin receptors (CLRs) [78]. PRRs primarily serve as a role in mediating inflammatory responses to endogenous and exogenous stimuli, including pathogen-associated molecular patterns (PAMPs) and DAMPs [79]. Ligand-receptor interactions activate downstream signals and engage in a series of physical reactions, such as inflammation, proliferation and carcinogenesis. For example, HMGB-1 interacts with TLR4 to induce expression of pro-inflammatory cytokines via activation of NF-κB, including tumor necrosis factor-α (TNF-α), IL-1, IL-6 [80]. However, there are some protective mechanisms preventing the effects of DAMPs, such as actin-scavenging system [75, 81]. Meanwhile, these factors often have a limited impact on local microenvironment instead of long-distance target tissues, owing to degradation and dilution in circulation [82]. Collectively, dying cells derived soluble factors are able to act as messages delivering specific signals via ligand-receptor interaction.

## Involvement of ghost messages and target cells in biological processes

Multicellular organisms maintain a dynamic homeostasis across varied cells and tissues through multiple signaling factors and transduction pathways [83]. Upon damage, a large number of cells die, release cellular contents and disturb the local tissue microenvironment. At the injury site, an adequate number of phagocytic cells clear cellular debris from damaged cells and senescent cells, releasing pro-resolution signals to prevent inflammation aggravation and progressive tissue necrosis, recover homeostasis and initiate remodeling [84, 85]. In addition, immune cells, stem and progenitor cells, stromal cells and resident tissue cells also receive these ghost messages to trigger an array of biological responses. In-depth understanding of these biological processes initiated by cell death derived signals may help us explain the etiology and pathogenesis of disease and develop new therapeutic strategies.

### Immune cells

Upon damage, immune cells are the first to respond to recognize, phagocytose, and present foreign antigens [86]. This coordinated process will initiate inflammation, with the ultimate goal of restoring to homeostatic state or set points [87].

Professional phagocytes, like macrophages are responsible for efferocytosis of apoptotic cells. Non-professional and specialized phagocytes are also involved in this process, including epithelial cells and Sertoli cells [88]. Generally, apoptosis is considered as immunologically silent without the release of DAMPs [89]. In addition, the removal of apoptotic cells by phagocytes is prompt and efficient, which activates anti-inflammatory pathways and promotes the secretion of transforming growth factor–β (TGF­β), IL-10 [90, 91], and long-chain fatty acid-derived lipids, such as lipoxin A4, and resolvins D1, D2 and E2 [92]. In turn, IL-10 and TGF-β can also increase efferocytosis by macrophages in a positive feedback loop [93]. This process is able to prevent DAMPs or auto-antigens from releasing into the surrounding environment, which is in part mediated by nuclear receptor 4A (Nr4a)1 [94]. Conversely, failure to appropriately clear (FAC) and failure to appropriately digest (FAD) by efferocytosis, proposed by Morioka et al*.* [95] and Trzeciak et al*.* [88] respectively, can induce a pro-inflammatory response. According to these reports, an increase in the level of injury and defects of efferocytosis lead to an accumulation of cell debris and auto-antigens which contribute to dramatic inflammation and autoimmunity [96]. For example, TNF-α has been shown to reduce the capacity for dead cell engulfment, which exacerbates inflammatory response [93]. A defective clearance of apoptotic cells and dsDNA antibody has been reported in systemic lupus erythematosus (SLE) [97]. Furthermore, when the digestion mechanisms are weak or overwhelmed, FAD will trigger the abnormal secretion of inflammatory factors, which make the organism be in a diseased state [88].

Efferocytosis also influences the metabolic state of macrophages, which promotes an anti-inflammatory response and continual efferocytosis [88]. During the onset of efferocytosis, apoptotic cells bind to PS receptors on phagocytes, which induce the increased expression of ABCA1 and SLC2A1, two metabolite transporters [88]. Efferocytosis increases intracellular contents and metabolic load, and degrades cellular debris into small molecules [92]. The metabolome of efferocytotic macrophages resembles an anti-inflammatory profile enriched in fatty acids and mitochondrial β-oxidation, electron transport chain, and increased coenzyme NAD + , rather than glycolysis [98]. Alternatively, some have reported upregulation of glycolysis-associated genes and downregulation of oxidative phosphorylation, fatty acid oxidation and de novo cholesterol synthesis associated genes [99]. Overall, these conflicting reports highlight the need for further elucidation of the metabolic profile of efferocytotic phagocytes.

In addition to the foregoing efferocytosis, PS is recognized by MerTK on immune cells, which blocks the release of inflammatory cytokines and initiates anti-inflammation response. For example, in four mouse organ injury models, it has been proved that MSCs transplantation treatment has shown the protective effect through spontaneous apoptosis of cells [100, 101]. Similarly, Zheng et al*.* showed that MSCs-derived apoptotic vesicles (apoVs) alleviated macrophage infiltration and re-established macrophage homeostasis in type 2 diabetes liver by virtue of surface signal CRT [102]. During apoptosis, caspase activation opens Pannexin 1 channels leading to AMP release [39]. As a “calm down” signal, AMP is converted to adenosine on macrophages, which activate anti-inflammatory genes Nr4a and Thrombospondin (Thbs)1 via A2a adenosine receptor [90]. There are some other immune cells engage in inflammation response. Lytic cell deaths released DAMPs also activate dendritic cells (DCs) and initiate adaptive T-cell immune responses [103]. Insufficient autophagy of deteriorated organelles leads to massive release of DAMPs, such as damaged mitochondrial, including mtDNA and mitochondria proteins [78]. Damaged mitochondria, an important source of reactive oxygen species (ROS), activate neutrophils through formyl peptide receptor-1 and TLR9 exacerbating the inflammatory response and modify the oxidative status of DAMPs [104].

Broadly speaking, immune cells participate in the balance between inflammation and homeostasis. Cues from the microenvironment of injured or regenerating tissue suggest that resolution of inflammation is crucial to initiate repair and chronic inflammation is always associated with diseases. Linking cell death and inflammation modulation of immune cells promotes the maintenance of homeostatic state, tissue regeneration and treatment of certain diseases.

### Stem and progenitor cells

Tissue damage and necrosis is generally followed by repair and regeneration, which requires stem and progenitor cell proliferation and differentiation. Stem and progenitor cells have the ability of self-renewal with a flexible differentiation potential, which enables them to replace senescent and damaged cells.

After injury, apoptotic cells release a variety of growth factors and cytokines under effector caspases, such as Wnt, bone morphogenetic protein (BMP), TGF-β, epidermal growth factor (EGF), fibroblast growth factor (FGF), insulin­like growth factor 1 (IGF-1) and Hedgehog mitogens signals to promote tissue homeostasis [105]. These factors mobilize and recruit stem and progenitor cells to the injury site, in which they proliferate, differentiate and restore tissue functions. In addition, lipid mediators also stimulate stem cell proliferation. For example, caspases activate calcium-independent phospholipase A_2_ (iPLA_2_) which produces arachidonic acid (AA). After cyclooxygenases (COX) and prostaglandin E2 (PGE2) synthase function, AA is converted into PGE2, a well-known stimulator of tissue regeneration [106, 107]. Moreover, HMGB1 accelerates tissue regeneration through HMGB1–CXCL12–CXCR4–G_Alert_ axis [108]. G_Alert_ is an intermediate state between G0 and G1, which enter the cell cycle rapidly than quiescent stem cells upon stimulation [108]. Except for soluble factors, cardiomyocyte-derived ApoBDs also facilitate proliferation and differentiation of cardiomyocyte precursors in vitro experiments [109]. Unfortunately, cancer stem cells are a subset of evil self-renewal cells with high tumorigenic potency. They are able to take advantage of the mechanism to cause tumor repopulation, greatly hindering tumor therapy [110].

Ghost messages also participate in stem and progenitor cell differentiation. Emerging evidence show that PS externalization influences cell differentiation processes for myotubes and osteoclasts, in which myoblasts fusion into myotubes and mesenchymal lineage osteoclast precursors fusion form multinucleated osteoclasts [111, 112]. During myoblast fusion, parts of myoblasts undergo apoptosis and PS externalization recognized by PS receptor BAI1, thus enhancing myoblast fusion through ELMO/Dock180/Rac1 signaling pathway [45]. In vitro experiments showed that blocking apoptosis inevitably impairs myoblast fusion in zVAD-treated cultures, and adding back apoptotic myoblasts rescued fusion, which entails cell–cell contact between apoptotic and viable myoblasts [113]. However, opposite views show that myoblasts fusing into myotubes are not undergoing apoptotic pathway [114], as adding exogenous PS liposomes significantly enhances myoblasts fusion into multinucleated myotubes [112]. Consequently, the fusion of myoblasts may merely be dependent on PS externalization in caspase-dependent manner.

During the early stage of osteoclastogenesis, PS externalization is necessary for M-CSF/receptor activator of nuclear factor κ-B ligand (RANKL)-induced fusion of pre-osteoclasts, and the process shares common PS receptors in the early fusion and late apoptosis [115]. Extracellular Anxs form a protein complex linking the PS-displaying cell surface with S100A4 or other protein molecules, which triggers osteoclast fusion [111]. However, PS exposure on the fusion-committed pre-osteoclasts correlates neither with activation of caspases 3 or 7 nor with characteristic features of apoptotic cells [111]. In contrast, one report showed that osteoclast precursors endocytose PS-containing liposomes, thereby secreting anti-inflammatory mediators TGF-β and PGE2, which in turn inhibit osteoclastogenesis and prevent trabecular bone loss by the downregulation of RANKL, RANK, intercellular cell adhesion molecule-1 (ICAM-1) and CD44 involved in the differentiation and fusion of osteoclast precursors [116].

Other mechanisms can promote cell differentiation. HUVEC derived ApoBDs initiate the differentiation of endothelial progenitor cells, which implies a role in angiogenesis and tissue healing [117]. In the process of osteogenic differentiation, MSCs engulf bone marrow apoptotic cells and take cues from efferocytosis, which enhance osteogenic differentiation [118]. In addition, HMGB1 also promotes MSCs to undergo osteogenic differentiation and promote lung fibroblasts differentiation into myofibroblasts, thus enhancing cell migration [119]. At low concentrations, TNF-α can lead to differentiation of satellite cells by activating p38 MAPK signaling [120]. IL-4 or IL-1β treatment can improve or inhibit muscle differentiation respectively, and blockade of IL-1β signaling significantly improves differentiation [121]. Taken together, receiving extracellular vesicles and cytokines from dying cells, stem and progenitor cells can modulate cell proliferation and differentiation.

### Stromal cells and resident tissue cells

Stromal cells and resident tissue cells are a population of important functional cells, which induce tissue regeneration and fibrosis. This process is essential for tissue repair after injury, but the role of ghost messages depends on the context.

Active communication between apoptotic cells and healthy cells promotes surrounding cells proliferation and maintenance of tissue homeostasis through what is commonly called as apoptosis-induced compensatory proliferation (Aip) [105]. This pathway of tissue regeneration is regarded as “Phoenix Rising”, which may rely on proapoptotic proteins (mostly caspases and other secretory factors) [106, 122]. The mechanisms of candidate factors inducing Aip have been studied in several model organisms, such as Drosophila, Hydra, Xenopus and mice [107]. For example, Atg1 in Drosophila controls regenerative proliferation after massive cell loss undergoing apoptosis [123]. Myo1D is necessary and sufficient for generation of ROS which promote Aip in the undead model [105].

Apoptotic cells derived EVs contain Wnt1, TGF-β, JNK, CrkI and miR-221/222, which promote proliferation in neighboring cells [124]. Proteomic analyses of these EVs show that upregulated proteins are annotated to function in cellular growth and proliferation processes, in metabolic processes and cytoskeletal and transport processes [82]. In addition, monocytes engulf dead neurons expressing osteopontin (OPN) and release exosome-like vesicles containing OPN. Following them, neurons and astrocytes carry out the regeneration and repair process [125]. ROS produced by dying hepatocytes induces release of IL-11, which triggers activation of JAK/STAT pathway in neighbor hepatocytes and compensatory proliferation [126]. IL-1 from necrotic hepatocytes caused by ROS also mediates Aip [127]. Cartilage homeostasis is regulated by dying cells derived pro-inflammatory cytokines, such as IL-6, IL-1β and TNF-α, which are responsible for catabolism and degradation of the cartilage [128, 129].

Combined, ghost messages provide a favorable or disadvantageous proliferation microenvironment for stromal cells and resident tissue cells, involving a dynamic regenerative process. Elucidating whether and how dying cells affect neighboring viable cells through ghost messages may shed light on the pathogenesis of certain diseases and develop potential treatments.

## Involvement of ghost messages in diseases

**Fig. 2:**
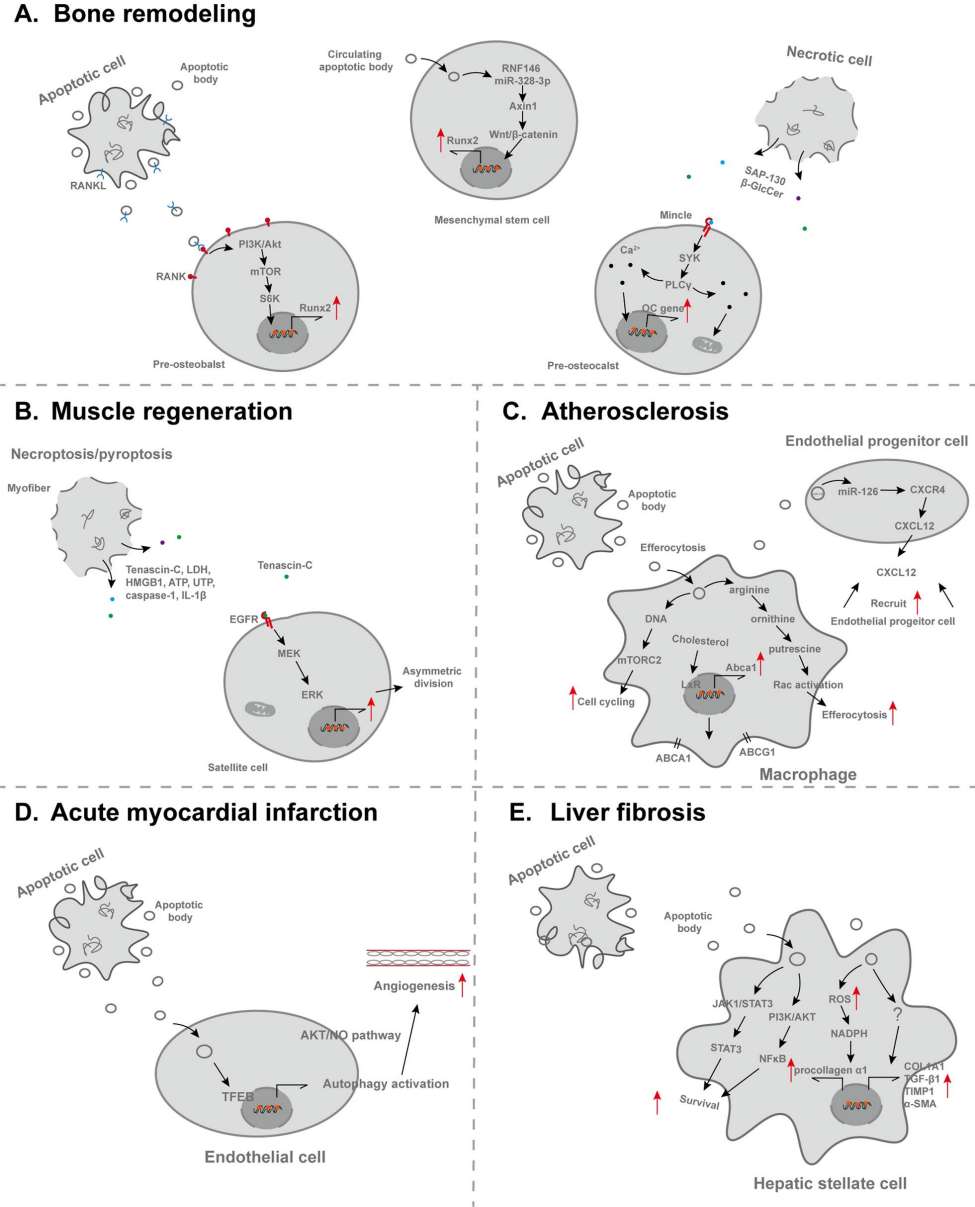
The involvement of ghost messages in diseases from different systems

Homeostasis and inflammation are two opposite physical states that are relevant to health and disease, respectively [87]. The process of cell death disturbs organism homeostasis, which if severe, will leave the body in a diseased state, for example systematic inflammation in the older people. As a result of the complexity of released molecules, ghost messages serve as a pluripotency role, involved in the development and progression of the disease [130] (Figure 2).

### Bone diseases

Cell death is typically involved in osteocytes, osteoblasts (OBs), osteoclasts (OCs), and chondrocytes which participate in bone remodeling, fracture and the pathogenesis of osteoporosis and osteoarthritis.

Osteogenic activity is carried out by an orchestrated system which involves mesenchymal lineage OBs-mediated formation coupled with hematopoietic lineage OCs-mediated resorption [131]. In OB-OC coupling, Osteoprotegerin/receptor activator of nuclear factor κ-B ligand/RANK (OPG/RANKL/RANK) signaling axis is one of the critical pathways, dysregulation of which leads to bone disorders, like osteoporosis [132]. RANKL reverse signaling can prevent bone from decreased formation by compensating for the loss of OB-OC coupling signals [133]. Mature OC derived ApoBDs enrich the high level of vesicular RANK, which binds to membranous RANKL of pre-osteoblasts to activate phosphoinositide 3-kinase/AKT Ser–Thr kinase/mammalian target of rapamycin/ribosomal protein S6 kinase (PI3K/AKT/mTOR/S6K) signaling pathway through RANKL reverse signaling and subsequently increase expression of osteogenic regulators Runt-related transcription factor 2 (Runx2) and Osterix (OSX) to promote osteogenic differentiation [134, 135]. Conversely, osteocyte derived ApoBDs are capable of inducing production of TNF-α by osteoclast precursors, thereby initiating osteoclastogenesis and local bone resorption [136]. More precisely, lncRNA *GM16222* and *E330032C10RIK* in ApoBDs play a regulatory role in angiogenic and osteogenic activities through acting as competing endogenous RNA (ceRNA) binding with relative miRNAs, such as miR-205-3p and miR-153-3p [34]. Local apoptosis is not limited, which can influence distant tissue, even the whole body. Circulating ApoBDs affect MSCs and ameliorate osteopenia by multiple cellular factors, such as ApoBDs-derived ubiquitin ligase RNF146 and miR-328-3p, which lead to Axin1 undergoing Poly-ADP-ribosylation (PARsylation) by Tankyrases 1 and 2 (Tnks1/2) and activate the Wnt/β-catenin signal. Subsequently, ApoBDs infusion improves the osteoporotic phenotype in a parabiosis mouse model [38].

After osteocytes undergo apoptosis, phagocytes cannot get access to and engulf apoptotic osteocytes in their isolated matrix. Therefore, these cells undergo secondary necrosis, resulting in membrane rupture and DAMPs release into extracellular environment, such as sin3A-associated protein 130 (SAP-130) and β-glucosylceramide (β-GlcCer)[137]. DAMPs activate PRR macrophage-inducible C-type lectin (Mincle) on osteoclasts, which leads to the activation of immunoreceptor tyrosine-based activation motif (ITAM)-based spleen tyrosine kinase (SYK)/calcium signaling pathways, priming osteoclast metabolic activity toward oxidative phosphorylation which is required for osteoclastogenesis [137]. HMGB1 protein is another widely reported cytokine acting to initiate tissue repair. When released by dying cells, HMGB1 binds to PRRs, such as TLRs on bone marrow stromal cells, promoting the release of TNF-α, RANKL and IL-6, but inhibiting the production of OPG, which further enhances osteoclastogenesis and remodeling of damaged bone [138].

ATP represents one of the most investigated DAMPs [65]. Extracellular ATP activates P2Y-GPCRs and P2X ligand-gated ion channels, both of which are expressed in osteocytes, osteoblasts and osteoclasts [139]. The activation of P2Y receptors enhances the expression of RANKL in osteoblasts, which leads to the increases of osteoclasts in the neighboring bone surface and enhanced bone resorption [140]. Meantime, Panx1-receptor is required for apoptotic osteocytes to trigger RANKL production in neighboring osteocytes [66]. In addition, neighboring cells can secrete mitogenic factors, like WNT, BMP, and EGF, which trigger cell proliferation [40]. Thus, the whole scenario should be apoptotic cells releasing ATP via Panx1 channels, thereby activating the secretion of RANKL and other factors from surrounding target cells, which engage in bone remodeling.

In summary, multiple death derived signals are involved in maintaining balanced OB-OC coupling, taking part in bone remodeling, explaining the pathogenesis and guiding clinical treatment.

### Skeletal muscle diseases

Skeletal muscle is an important locomotive and endocrine organ with active metabolism. Trauma, aging and disease are concomitant with skeletal muscle injury and loss, which impair muscle function. But skeletal muscle has the ability of regeneration after injury. The process of regeneration is extremely complex and muscle regeneration microenvironment includes diversified cellular populations and cytokines, such as myocytes, satellite cells, endothelial cells, fibroblasts, mesenchymal stem cells and immune cells, as well as various factors from secretion or circulation [141]. Satellite cells are indispensable for muscle regeneration, modulated by interplay between intrinsic factors within satellite cells and extrinsic factors in microenvironment [142].

After muscle injury, myofibers commit MLKL-dependent necroptosis and gasdermin-dependent pyroptosis, sharing a common pro-inflammatory feature that cellular membrane ruptures, thereby releasing HMGB1, ATP, UTP, LDH, caspase-1, IL-1β and NLRP3 inflammasome proteins into the extracellular space [143, 144]. Necroptosis is required for proper muscle regeneration, either directly removing a subpopulation of potentially harmful muscle stem cells undergoing epigenetic rewiring, or promoting satellite cell proliferation or recruiting immune cells via some molecules [143]. Zhou et al*.* found that necroptotic myofibers released Tenascin-C (TNC) to facilitate satellite cells proliferation. TNC contains an EGF-like domain and can serve as an EGF mimic to activate EGFR signaling pathway, involving EGFR, MEK, and ERK phosphorylation to increase the asymmetric division of satellite cells. Damaged-myofiber-derived factors, such as metabolic enzymes glyceraldehyde-3-phosphate dehydrogenase (GAPDH) stimulates satellite cells transit from the G0 to the G1 stage, thereby promoting activation and expansion of satellite cells through the BMP signaling pathway [145]. In vivo, prior to muscle injury, the treatment of recombinant GAPDH expands the satellite cell population [145]. In addition, all kinds of pro-inflammatory factors can recruit neutrophils, macrophages, regulatory T cells and dendritic cells, which seems to disturb the microenvironment, but muscle satellite cells fail to activate the regenerative potential in the absence of the inflammatory response [146]. For instance, macrophage polarization to M2 phenotype promotes revascularization and muscle regeneration in hindlimb ischemia models [147].

Pyroptosis is a kind of gasdermin-mediated programmed necrotic cell death in response to certain bacterial insults, marked by the formation of inflammasome, activation of caspase-1 and release of proinflammatory cytokines IL-1β and IL-18 [148, 149]. It is generally viewed as an inflammatory form of cell death, a process regulating cellular differentiation and proliferation [150]. Pyroptosis of muscle cell is concomitant with the expansion of neighboring muscle cells, which depends on IL-1β secretion as well as its downstream mediator of muscle hyperplasia, IGF-1 [144]. IL-1β has been shown to increase the proliferation of skeletal muscle satellite cells, promote NF-κB activity and involve COX-2-dependent prostaglandin (PGs), which is a kind of lipid mediator involved in skeletal muscle regeneration [151]. Contrastingly, a report from Cohen et al*.* showed that inhibitory effects of M1 macrophages on myogenesis were mediated by IL-1β signals, implicating IL-1β as the factor accounting for attenuated muscle regeneration in dysferlin-deficient muscle [121]. Taken together, the role of inflammatory cytokines may be different and context dependent. Thus, during muscle regeneration, signal transduction between dying cells and viable cells is dynamic and complicated. Targeting these ghost messages and interpreting the role in muscle regeneration will lead to develop of new drugs against skeletal muscle diseases, such as sarcopenia and Duchenne muscular dystrophy (DMD).

### Cardiovascular diseases

Coronary artery is an important source of perfusion to the heart. When atherosclerosis happens in coronary artery with vessel lumen stenosis, the patients are likely to suffer from acute myocardial infarction due to the sudden interruption of blood flow [152]. According to recent epidemiological data, atherosclerotic cardiovascular disease and myocardial infarction are considered as global public health issues and remain the leading cause of cardiovascular-related deaths [153].

#### Atherosclerosis

A convincing body of data shows that inflammation participates fundamentally in the pathophysiology of atherogenesis [6]. Atherosclerotic plaque is the principal pathological change characterized by its necrotic core, fibrous cap and foam cells [153].

Apoptotic cells release chemokinetic cytokines to stimulate plaque inflammation [154], but efferocytosis is a crucial anti-inflammation process. Macrophages engulf apoptotic cells during efferocytosis and take up arginine and ornithine, which are converted into putrescine [155]. Putrescine strengthens subsequent efferocytosis by increasing Rac1 activation and promotes resolution of atherosclerosis [155]. Meantime, apoptotic cell-derived nucleotides trigger proliferation of efferocytosing macrophages through the DNA-PKcs-mTORC2/Rictor-Myc pathway [156]. In addition, phagocytes take up large quantities of cholesterol as well [157]. Efferocytosing macrophages efficiently upregulate the cholesterol transporters ATP-binding cassette transporters ABCA1 and ABCG1 in BAI1/ELMO1/Rac pathway, thus enabling efflux of intracellular cholesterol to form the lipid-rich high-density lipoprotein (HDL) [83, 157]. Impairments in cholesterol efflux are associated with dyslipidemia and atherosclerosis [157]. Phagocytes also increase cell-surface glucose transporter SLC2A1, which increases glucose uptake, promotes glycolysis and enhances subsequent efferocytosis [95]. Chronic inflammation is concomitant with defective efferocytosis, which cause a series of adverse outcomes [154]. Impaired efferocytosis results in the accumulation of dying cells or cell debris, thus gradually promoting formation of necrotic lipid core of the atherosclerotic plaque, contributing to plaque expansion and reduced plaque vulnerability, leading to plaque disruption and tissue ischemia or infarction [158]. It is known that OxLDLs directly compete with ApoBDs for engulfment receptors and lead to inefficient efferocytosis [159]. Kojima et al*.* reported that CD47 blocking antibodies reversed the defect of efferocytosis and ameliorated atherosclerosis [160]. Moreover, vascular smooth muscle cells (VSMCs) and endothelial cells play a part in progression of atherosclerosis. During early plaque growth, ApoBDs recruit endothelial progenitor cells to repair the damage [161]. Endothelial cell–derived ApoBDs carry microRNA-126 (miR-126) to mediate the atheroprotective effects. MiR-126 upregulate the expression of CXCR4 and CXCL12 to recruit progenitor cells [161]. Similarly, VSMCs–derived ApoBDs also mediate secretion of CXCL12 [162]. Other in vivo experiments show that inducible VSMC apoptosis promotes cell proliferation after vessel injury [163]. Understanding the above mechanisms, Wu et al. showed that the use of apoptotic body biomimic liposome primed M2 macrophages polarization and promoted an anti-inflammatory response in atherosclerotic plaques [164]. Blockade of inflammatory factors IL-1β decreased the necrotic core, increased fibrous cap thickness and enhanced plaque stability [165].

#### Acute myocardial infarction

Acute myocardial infarction (AMI) is accompanied by vascular trauma, myocardial cell death, and ultimate fibrotic repair. Vascular trauma in AMI is followed by vascular cells apoptosis and a rapid mobilization of endothelial progenitor cells [117]. Local application of MSC-derived ApoBDs activates macroautophagy/autophagy pathway in recipient endothelial cells, promoting the expression of transcription factor EB (TFEB) and autophagy-related gene. This in turn promotes endothelial cell proliferation, enhances angiogenesis and improves cardiac functional recovery via AKT-NO pathway [166]. In addition, AMI contributes to cardiomyocytes apoptosis and necrosis, releasing DAMPs, such as HMGB1. HMGB1 interacts with TLR9 to control myocardial inflammation and improve harmful outcomes [167]. This is supported by cardiac-specific overexpression of HMGB1 or local treatment of HMGB1, that showed enhanced angiogenesis and myocardial regeneration [168, 169]. Furthermore, DAMPs trigger fibroblast proliferation and motility, and collagen mRNA expression in a TLR4- and RAGE-dependent manner [170].

Combined, ghost messages partially explain the development and prognosis of atherosclerotic cardiovascular disease and AMI. Further efforts are needed to understand the underlying molecular mechanism of ghost messages and provide experimental evidence for developing potential targeted treatments.

### Hepatic disease

Liver is a powerful metabolic and secretory organ, which is able to metabolize drugs and alcohol, cope with exhausted cells and is often irritated by various substances [93]. Long-term exposure to harmful substances, there are a lot of damaged hepatocytes involving in viral hepatitis, alcoholic and non-alcoholic steatohepatitis (ASH and NASH), and drug-induced liver injury (DILI) [171].

Dying cells derived non-invasive biomarkers are becoming promising tools to manage and diagnose liver injury in various acute and chronic liver diseases [172]. For example, during hepatocytes death, cytokeratin-18 (CK18) is released into the extracellular environment [172]. Hence, CK18 has been listed as a biomarker in the latest practice guidance for diagnosis and management of nonalcoholic fatty liver disease (NAFLD) [173]. In addition, soluble Fas ligand, TNF-α and TNFR levels are increased in hepatitis, which are expected to be interesting targets to monitor disease progression [174]. In addition, microRNA-122 is a liver specific miRNA, presented in hepatocytes and released into circulation after hepatocytes damage [175]. Several other miRNAs are also elevated in patients with NAFLD, such as miR-192, miR-21, miR-34a, and miR-451 [176].

An important pathological feature of liver disease is liver fibrosis, which is mainly mediated by hepatic stellate cells (HSCs) and pro-inflammatory Kupffer cells [177]. Hepatocyte-derived ApoBDs ingestion promotes HSCs survival via the activation of JAK/STAT and PI3K/AKT/NF-κB cascade, which upregulate anti-apoptotic protein Mcl-1 [5]. The engulfment of ApoBDs by HSCs is profibrogenic, as evidenced by upregulated profibrotic genes, such as COL1A1, TGF-β1, TIMP1, TIMP2 and α-SMA, and a myofibroblast morphology with collagen producing [178]. Interestingly, although TGF-β is an anti-inflammatory cytokine, it is profibrogenic in liver [179]. In chronic hepatitis C, ApoBDs contain the nonstructural hepatitis C proteins, which activate HSCs and promote fibrosis and cirrhosis [180]. Moreover, phagocytosis of ApoBDs increases ROS, which activates nicotinamide adenine dinucleotide phosphate reduced (NADPH) oxidase, thus leading to upregulation of procollagen α1(I) [181]. In addition to apoptosis, hepatocytes undergo necroptosis, pyroptosis and ferroptosis, which trigger robust inflammatory responses, thus recruiting immune cells and activating HSCs [11]. For example, hepatocytes undergoing pyroptosis release NLRP3 inflammasome proteins and activated caspase-1 [182]. Growing evidence shows that NLRP3 inflammasome activation is an important driver of various liver diseases, namely inflammasome-driven fibrogenesis [183]. HSCs engulfing NLRP3 inflammasome lead to the increased expression of IL-1β and α-SMA. Besides, activated caspase-1 is detected in patients with NASH and promises to be a diagnostic biomarker [182].

Liver possesses a specific regenerative capacity [184]. Following partial hepatectomy, production of PGE2 in caspase-3 dependent manner contributes to liver regeneration. Released ATP is also an important regenerative factor for hepatocytes via P2Y_2_ receptor [185]. Dying hepatocytes release ROS, which activates ERK and stimulates Fra-1 phosphorylation, thus increasing IL-11 expression [186, 187]. IL-11 triggers the STAT3 phosphorylation ultimately leading to apoptosis-induced compensatory proliferation (Aip) [187]. In chronic liver diseases, loss of hepatocytes and a persistent inflammatory microenvironment result in abnormal liver cell proliferation [188], culminating in the occurrence and development of hepatocellular carcinoma [179].

Overall, continuous inflammation and proliferation contribute to the progression of hepatitis to cirrhosis and liver cancer, but whether effective control of inflammation can delay the development of the disease remains to be explored. Moreover, developing novel non-invasive diagnostic biomarkers will also be the focus of future research.

### Tumors

**Fig. 3:**
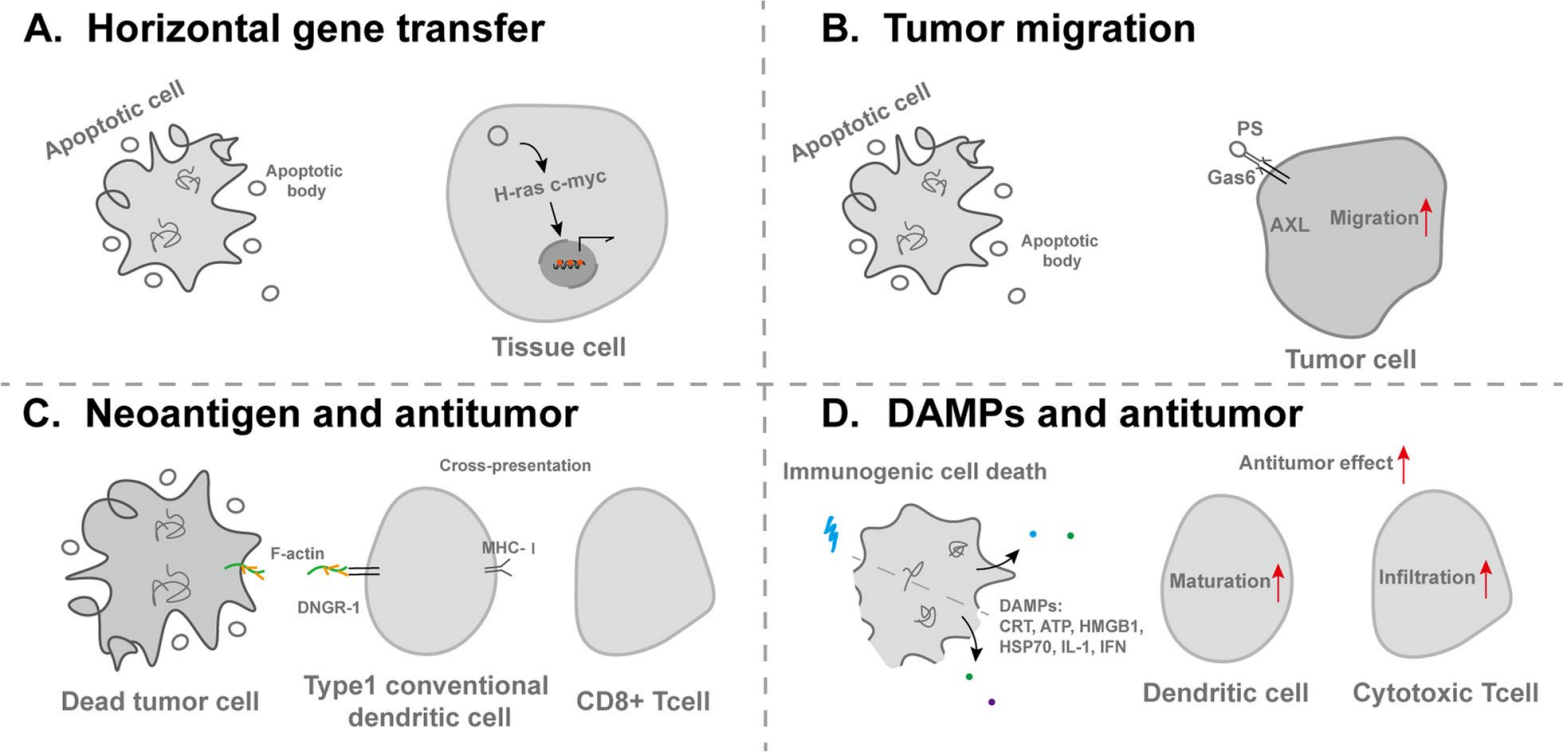
The involvement of ghost messages in tumor

Tumor is product of abnormal regeneration in essence. The onset of tumors is extraordinarily complex, reflected in the intricate inducements and pathogenesis. In clinical frontline treatments, chemotherapy, radiotherapy and immunotherapy are designed to induce tumor cell death and suppress tumor growth [189]. Meantime, because of hypoxia, low pH and the lack of nutrition, large amounts of tumor cells die in the course of solid tumor growth. It is estimated that apoptotic cells account for 70% of the tumor cell population in glioblastoma[190], suggesting that a number of ghost messages will be released into the tumor microenvironment (TME) to affect viable cell phenotypes, such as malignant transformation, proliferation, migration, drug resistance, inflammation and cell death [191, 192] (Fig. 3).

Dying tumor cells release numerous bioactive molecules, part of which serve as growth signals, such as ROS, IL-6 and caspase-3. By virtue of Aip, these growth signals induce proliferation and repopulation of remaining cells including cancer stem cells, treatment-resistant cells, surviving tumor cells and stromal cells, potentially limiting the effectiveness of anti-cancer drugs and causing poor prognosis, such as advanced breast tumors [193]. Meantime, growth signals, such as VEGF-α, also stimulate angiogenesis and neovascularization of tumor in gliomas xenograft model, which promotes gliomas growth and progression [194]. Moreover, tumor cell efferocytosis is often immunosuppressed with increased immunosuppressive cytokines IL-4 and IL-10, myeloid-derived suppressor cells and regulatory T cells in mammary tumor mice model, which are accompanied with weak antitumor immune response [195].

Dying tumor cells released molecules usually affect local inflammation reaction in TME. Chronic inflammation states are usually tumor-promoting, characterized by constant death and incomplete recovery, which predispose to the development of certain tumors, such as colorectal cancer and hepatic cellular carcinoma [196, 197]. However, antitumor immune response is crucial for tumor treatment and poor anti-cancer immune response are relevant to adverse outcomes. In “cold tumors”, such as diffuse intrinsic pontine glioma, limited immune cell infiltration, reduced inflammatory factors secretion and rare antigen presenting cells limit the effects of immunotherapy [198]. Currently, one promising way is to activate tumor immune microenvironment by inducing immunogenic cell death, which transforms “cold” immunosuppressive TME to “hot” immunoresponsive TME [199]. Some antitumor therapies, such as oncolytic viruses, photodynamic therapy, radiotherapy and certain chemotherapeutic drugs, can induce the immunogenic cell death, which releases tumor antigens and DAMPs, such as CRT, HSP70, HSP90, HMGB1, IL-1 and type I IFN, thereby robustly activating the innate and adaptive immune systems by facilitating maturation of DCs and infiltration of cytotoxic T lymphocytes [200–202]. Researches have shown the potential applications of immunogenic cell death inducers in tumor treatment. For example, near‑infrared photoimmunotherapy induced cell death enhances DCs migration via ATP-P2X7 and HMGB1-TLR4 pathway in colon adenocarcinoma-bearing mice [203]. In addition, immunogenic cell death inducer oxaliplatin has been widely used in the treatment of colorectal, gastric, and pancreatic cancers [204]. Consequently, treatments aiming at immunogenic cell death targets and strengthening antitumor immune response will greatly improve clinical outcomes. For example, the use of cytotoxic T lymphocyte protein 4-blocking antibodies strengthens adaptive immune response and induces a survival benefit in patients with advanced melanom [201]. In addition to cytokines, necrotic tumor cells derived neoantigens also produce an antitumor effect. For example, neoantigens associate with F-actin cytoskeleton, which binds C-type lectin receptor DNGR-1 highly expressed on DCs. Through cross-presenting antigens with MHC class I molecules, the process strengthens anti-cancer effects of CD8^+^ T cells [75].

Dying cell derived EVs in TME are another important ghost messages, whereas limited studies elaborate the relationship between dying cell derived EVs and tumor, because of complex EVs composition in TME. Researches show that oncogenes from tumor cells may be horizontally transferred by ApoBDs, such as H-ras and c-myc, as well as drug resistance genes, which will result in accumulation of genetic changes required for neoplasia and recurrence [205]. For example, surrounding fibroblasts engulf lymphoma-derived ApoBDs, thereby resulting in the integration of lymphoma-derived DNA into the fibroblast genome [206]. Meantime, apoptotic EVs promote a more aggressive and therapy-resistant glioblastoma via the transfer of spliceosomal proteins and small uridine-rich non-coding RNAs to alter RNA splicing [190]. In addition, apoptotic tumor cell-derived EVs promote tumor growth and metastasis in the onco-regenerative niche [207]. For example, ApoBDs enhance the migration of tumor cell lines through PS-Gas6-AXL signaling pathway [208]. Moreover, chemotherapy and hypoxia induced tumor cell released microparticles inhibit inflammation and promote progression and metastasis [209]. Interestingly, radiated tumor cell–released microparticles exhibit broad antitumor effects and promote macrophage polarization in a mouse model of malignant pleural effusion [210]. Also, MSC-derived apoEVs facilitate Fas trafficking to cell membrane and induce multiple myeloma cell apoptosis [211]. In general, the role of dying cells derived EVs in tumor is complicated and indistinct, and needs further exploration.

Based on the characteristics of ghost messages, curative vaccine and adjuvant are developed using antigen presenting cells that have phagocytosed ApoBDs or tumor cells lysates, which generates a tumor-specific cytotoxic T-cell response in the immunotherapy of tumors and will be a useful treatment to eradicate tumor cells [212, 213]. In addition, some engineered vesicles have also been developed to treat tumors. Liu et al*.* show that the matrix metalloproteinase 2-sensitive ApoBD-mimicking nanoparticles loaded with dasatinib significantly delete tumor-associated macrophages and improve anticancer activity in breast tumor-bearing mice model [214]. Gao et al*.* provide evidence that the infusion of apoptotic tumor cells derived methotrexate-containing plasma-membrane EVs into bile-duct lumen of patients with extrahepatic cholangiocarcinoma relieve biliary obstruction [215]. Another group find that lymphocytes generated vesicle-like ApoBDs containing anti-neoplastic drug protoporphyrin X deliver therapeutic drugs for Ras-mutated breast cancer cells [216]. ApoBDs also carry the remaining drugs from parental cells to neighboring tumor cells, thus contributing to the efficient intercellular drug delivery [217]. Other studies have reported that the intravenous injection of ApoBDs-encapsulated nanomedicine can be engulfed by inflammatory monocytes, which infiltrate into the tumor center and efficiently ablate tumors in EL4 tumor-bearing mice [218].

To sum up, the occurrence and development of tumors are extremely complicated. Developing novel treatments is urgent, and immunogenic cell death and vesicles-based therapeutic strategies have shown great potential.

## Conclusion

Intercellular “ghost messages” are classified as extracellular vesicles and soluble factors, which influence the local microenvironment, perform important physiological functions and steer the body in a good or bad direction. As candidate biomarkers, potential drug carriers, growth factors and anti-inflammation molecules, their devotion can be described as Chinese ancient poems “Falling is not heartless things, into the mud more flowers”. But they can also show a negative side, such as engaging in chronic inflammation, detrimental proliferation and tumorigenesis. Though this regulative network is complicated, the development of high-throughput techniques (e.g., proteomics, spatial transcriptomics, and metabolomics) has provided us some cues to investigate the relationship between cell death signals and pathogenesis in terms of molecular and signaling pathways, which has aroused wide attention. Based on this, we enable to develop novel treatments relying on soluble factors and EVs.

Although part of ghost messages and their targets have been summarized in this review, as well as induced effects, there are still many open questions remain unanswered. Firstly, will recipient cells receive “crasher uninvited guest” actively or passively and which cells will receive these signals. These will require single-cell sequencing and spatial transcriptome analysis to answer. Secondly, is the intercellular communication led by ghost messages an essential way of normal cellular function or a stress response to injury factors? What is the extent of the cellular response, causing physiological or pathological effects? Next, the mechanism of cargos sorting, characterization, target receptors, specific recognition modes, biodistribution and systemic effects of extracellular vesicles need to be systematically studied. Lastly, how will we develop clinical disease interventions, aiming at cell death or ghost messages and its receptors, which will provide insights into present and future treatments. It is believed that in the near future, more biologics and ghost messages-based therapies will be used for clinical benefit.

Cell death is classified as nonlytic cell death and lytic cell death based on their morphologic feature, which is membrane rupture and cellular contents release. In the process of cell death, dying cells can release large amounts of soluble factors and extracellular vesicles. These soluble factors include damage-associated molecular patterns (DAMPs), autoantigens and cytokines, which are secreted actively via channels or vesicle transportation, or released passively through GSDMD pore/GSDME pore/MLKL pore or broken membrane. Extracellular vesicles consist of apoptotic bodies, exosomes and microvesicles with diversified sizes, which are secreted from multivesicular endosomes or shed from plasma membrane, carrying proteins, nucleic acids and lipids. These ghost messages act as signal molecules or carriers, which are transported to local or remote microenvironment to exert effects on healthy cells. The recipient cells endocytose soluble factors and vesicles, or these ghost messages interact with receptors or channels, or fuse directly with plasma membrane. Subsequently, these ghost messages deliver the information into the recipient cells to initiate a series of biological process, which are potentially involved in the occurrence and progress of diseases.

(A) ApoBDs enriching the high level of RANK bind to membranous RANKL of pre-osteoblasts to activate PI3K/AKT/mTOR/S6K signaling pathway through RANKL reverse signaling and increase the expression of osteogenic regulators Runx2. Circulating ApoBDs-derived RNF146 and miR-328-3p lead to Axin1 undergoing PARsylation and activate the Wnt/β-catenin signal. Necrotic cells release DAMPs, such as SAP-130 and β-GlcCer, which activate Mincle on osteoclasts, leading to the activation of SYK/calcium signaling pathways, thus promoting oxidative phosphorylation and the transcription of osteoclast genes. (B) Necroptotic or pyroptotic myofibers release Tenascin-C, LDH, HMGB1, ATP, UTP, caspase-1 and IL-1β into the extracellular space. For example, Tenascin-C can serve as an EGF mimic to activate EGFR signaling pathway, involving EGFR, MEK, and ERK phosphorylation to increase the asymmetric division of satellite cells, which contribute to muscle regeneration. (C) Macrophages engulf apoptotic cells during efferocytosis and take up arginine and ornithine being converted into putrescine, which strengthens subsequent efferocytosis by increasing Rac1 activation and promotes resolution of atherosclerosis. Apoptotic cell-derived nucleotides trigger proliferation of efferocytosing macrophages through the DNA-mTORC2 pathway. Efferocytosing macrophages efficiently upregulate the cholesterol transporters ABCA1 and ABCG1 enabling efflux of intracellular cholesterol. Endothelial progenitor cells phagocytose ApoBDs carrying miR-126 to mediate the atheroprotective effects by upregulating the expression of CXCR4 and CXCL12 to recruit progenitor cells. (D) Endothelial cells phagocytose ApoBDs to promote the expression of TFEB and autophagy-related genes, thus enhancing angiogenesis and improving cardiac functional recovery via AKT-NO pathway. (E) ApoBDs promote HSCs survival via the activation of JAK/STAT and PI3K/AKT/NF-κB cascade. Meantime, the engulfment of ApoBDs by HSCs is profibrogenic, accompanied by upregulated profibrotic genes, such as COL1A1, TGF-β1, TIMP1 and α-SMA. In addition, phagocytosis of ApoBDs increases ROS, which activates NADPH oxidase, thus leading to upregulation of procollagen α1.

(A) Oncogenes from tumor cells can be horizontally transferred by ApoBDs, such as H-ras and c-myc. (B) ApoBDs enhance the migration of tumor cells through PS-Gas6-AXL signaling pathway. (C) Neoantigens F-actin cytoskeleton on dying tumor cells binds DNGR-1 highly expressed on type1 conventional dendritic cells, which strengthens anti-cancer effects of CD8 + T cells through cross-presenting antigens with MHC-I molecules. (D) Some antitumor therapies induce the immunogenic cell death, which releases DAMPs, such as CRT, ATP, HMGB1, HSP70, IL-1 and IFN, thereby strengthening the antitumor immune effects by facilitating maturation of dendritic cells and infiltration of cytotoxic T lymphocytes.

## Supplementary Information

**Additional file 1**: **Table S1**. Details of dying cell derived extracellular vesicles in various diseases. **Table S2**. Details of engineering dying cell derived extracellular vesicles in various diseases.

## Data Availability

All data generated or analysed during this study are included in this published article and its supplementary information fles.
