# Supplementary material for: Ghost messages: cell death signals spread
Source: Cell Commun Signal. 2023 Jan 9;21:6. doi: 10.1186/s12964-022-01004-0 (PMC9830882; doi:10.1186/s12964-022-01004-0)
Supplement: Supplementary file 2 — Additional file 1: Table S1. Details of dying cell derived extracellular vesicles in various diseases. Table S2. Details of engineering dying cell derived extracellular vesicles in various diseases. [file 12964_2022_1004_MOESM2_ESM.docx]

**Supplementary table 1.** Details of dying cell derived extracellular vesicles in various diseases.

| EVs types | Type of cell death | Size | Parental cell | Target cell | Cargo | Experimental model | Effects | Mechanism |
| --- | --- | --- | --- | --- | --- | --- | --- | --- |
| ApoEVs | apoptosis | 50-500nm | glioblastoma cells | glioblastoma cells | spliceosomal proteins and small non-coding RNAs | intracranial xenograft tumor model | epithelial to mesenchymal transition, growth of glioblastoma cells and therapy resistance | mRNA alternative splicing[1] |
| ApoBDs | apoptosis | 1 000-3 000nm | macrophages | lung epithelial cells | microRNAs (miR-221/222) | G^-^ bacterial pneumonia model | proliferation of lung epithelial cells | cyclin-dependent kinase inhibitor 1B (CDKN1B) pathways[2] |
| ApoEVs | apoptosis | 50-250 nm | mesenchymal stem cells | multiple myeloma cells | Fas-ligand (FasL) | multiple myeloma model | initiation of apoptosis and inhibition of multiple myeloma cell growth | Fas trafficking and FasL/Fas pathway[3] |
| ApoBDs | apoptosis | 1 000-2 000nm | MDA-MB-231 cells and SK-MES-1 cells | MDA-MB-231 cells and SK-MES-1 cells | PS | —— | cancer cell migration | PS-Gas6-AXL signaling axis[4] |
| ApoEVs | apoptosis | 50-600nm | mesenchymal stem cells | macrophages | CRT | type 2 diabetes mice | macrophage polarization and inhibition of macrophage accumulation | CRT-mediated eﬀerocytosis[5] |
| ApoEVs | apoptosis | 50–100 nm | thymocytes | macrophages | PS | colitis model | Tregs differentiation and inhibition of Th1 cell response | PS-FOXO3-TGFβ pathway[6] |
| Microparticles | apoptosis | <1 000nm | platelets | macrophages | PS and integrins | —— | macrophage polarization and inhibition of proliferation | integrins-mediated adhesion and endocytosis[7] |
| Apoptotic exosome-like vesicles | apoptosis | 30-100nm | endothelial cells | T cells and B cells | 20S proteasome core | allografted mice model | production of autoantibodies and acceleration of rejection | proteasome activity[8] |
| ApoBDs | apoptosis | —— | preosteoclasts and mature osteoclasts | endothelial progenitor cells and mesenchymal stem cells | PDGF-BB and RANK | calvarial bone defect model | enhanced angiogenesis and osteogenesis | growth factor and RANKL reverse signaling[9] |
| Microparticles | pyroptosis | —— | THP1 monocytic cells | endothelial cell | active caspase-1 and cleaved GSDMD | —— | endothelial cell apoptosis | caspase-1 and GSDMD induced cell death[10] |
| ApoBDs | apoptosis | —— | lymphocytes | 4T1 cells | protoporphyrin X (PpIX) | 4T1 tumor bearing mice | cancer cell apoptosis | PpIX produce ROS and kill the cancer cells under the light irradiation[11] |
| ApoBDs | apoptosis | —— | 4T1 cells and 3T3 cells | 4T1 cells | camptothecin and PR104A | 4T1 tumor bearing mice | enhanced drug penetration and tumor destruction | neighboring effect and cytotoxicity[12] |
| ApoBDs | apoptosis | 1 000-5 000nm | lymphoma cell line EL4 | monocytes/macrophages | CpG modified AuNR | EL4 tumor bearing mice | tumor ablation and enhanced immunity | natural tumor-homing, nanorod-based photothermal effect and CpG-promoted immunostimulation[13] |
| Microvesicles | apoptosis | —— | HeLa cells | HeLa cells | CrkI | nephrotoxic serum–induced glomerulonephritis animal model | increased cell proliferation | activation of c-Jun N-terminal kinase (JNK)[14] |
| Small ApoBDs | apoptosis | <1 000 nm | B16F10 cells | microglial cells | anti-TNF-α antisense oligonucleotide (ASO) | Parkinson’s disease mouse model | reduced inflammation and ameliorated symptoms | CD44v6-mediated penetrating the blood-brain barrier and ASO-induced anti-inflammation[15] |
| ApoBDs | apoptosis | —— | hepatocytes | hepatic stellate cells (HSCs) | nonstructural HCV proteins | —— | amplified fibrogenic mRNA expression and enhanced HSC activation | PS and HCV proteins-mediated activation[16] |
| Ferroptosis-dependent Evs | ferroptosis | 150-350nm | THP-1 macrophage | mesothelial cells | ferritin and iron | asbestos-induced peritonitis model | genomic damage and carcinogenesis | iron induced oxidative DNA damage[17] |
| ApoEVs | apoptosis | 50-200nm | mesenchymal stem cells | mesenchymal stem cells | —— | MRL/lpr mice, cutaneous wound healing model and  dorsal hair regeneration model | improved skin and hair follicle MSCs, promotion of cutaneous wound healing and hair regeneration | activation of Wnt/β-catenin pathway[18] |
| Abbreviations: EVs, extravcllular vesicles; apoEVs, apoptotic EVs; ApoBDs, apoptotic bodies; PS, phosphatidylserine; CRT, calreticulin. | | | | | | | | |

**Supplementary table 2.** Details of engineering dying cell derived extracellular vesicles in various diseases.

| EVs types | Type of cell death | Size | Parental cell | Target cell | Cargo | Experimental model | Effects | Mechanism |
| --- | --- | --- | --- | --- | --- | --- | --- | --- |
| Chimeric apoptotic bodies | apoptosis | 1 000nm | T cells | macrophages | apoptotic bodies membrane, mesoporous silica nanoparticles and microRNA-21 or curcumin | cutaneous wounds and colitis models | M2 polarization, promote regeneration | natural membrane and microRNA­21 or curcumin-mediated anti-inflammation[19] |
| Nanoparticles | apoptosis | 105-126nm | NIH 3T3 fibroblasts | bone marrow-derived macrophages | PS and a poly(lactide-co-glycolide) core | —— | macrophage polarization | natural membrane-mediated anti-inflammation[20] |
| Microvesicles | apoptosis | —— | human tumor cells | neutrophils | methotrexate-containing plasma-membrane | patients with extrahepatic cholangiocarcinoma | activated neutrophils and relieved biliary obstruction | microvesicles induced pyroptosis through a gasdermin E-dependent pathway[21] |
| Nanoparticles | apoptosis | 230nm | —— | tumor-associated macrophages (TAMs) | PS and dasatinib | breast tumor-bearing mouse model | depletion of TAMs and improved anticancer activity | PS and drug-mediated[22] |
| Apoptotic body biomimic liposome | apoptosis | 90-140nm | —— | macrophages and human umbilical vein endothelial cells | PS, DSPE-PEG2000-cRGDfK and pioglitazone | in vitro atherosclerotic plaque models | macrophage polarization and slow progression of atherosclerosis | natural membrane and peroxisome proliferator-activated receptor γ agonist[23] |
| Abbreviations: PS, phosphatidylserine. | | | | | | | | |

**References**

1. Pavlyukov MS, Yu H, Bastola S, Minata M, Shender VO, Lee Y, Zhang S, Wang J, Komarova S, Wang J, et al. Apoptotic Cell-Derived Extracellular Vesicles Promote Malignancy of Glioblastoma Via Intercellular Transfer of Splicing Factors**.** Cancer Cell. 2018; 34(1)**:**119-135.e10.

2. Zhu Z, Zhang D, Lee H, Menon AA, Wu J, Hu K, Jin Y. Macrophage-derived apoptotic bodies promote the proliferation of the recipient cells via shuttling microRNA-221/222**.** J Leukocyte Biol. 2017; 101(6)**:**1349-1359.

3. Wang J, Cao Z, Wang P, Zhang X, Tang J, He Y, Huang Z, Mao X, Shi S, Kou X. Apoptotic Extracellular Vesicles Ameliorate Multiple Myeloma by Restoring Fas-Mediated Apoptosis**.** Acs Nano. 2021.

4. Zweemer AJM, French CB, Mesfin J, Gordonov S, Meyer AS, Lauffenburger DA. Apoptotic Bodies Elicit Gas6-Mediated Migration of AXL-Expressing Tumor Cells**.** Mol Cancer Res. 2017; 15(12)**:**1656-1666.

5. Zheng C, Sui B, Zhang X, Hu J, Chen J, Liu J, Wu D, Ye Q, Xiang L, Qiu X, et al. Apoptotic vesicles restore liver macrophage homeostasis to counteract type 2 diabetes**.** Journal of Extracellular Vesicles. 2021; 10(7).

6. Chen H, Kasagi S, Chia C, Zhang D, Tu E, Wu R, Zanvit P, Goldberg N, Jin W, Chen W. Extracellular Vesicles from Apoptotic Cells Promote TGFβ Production in Macrophages and Suppress Experimental Colitis**.** Sci Rep-Uk. 2019; 9(1).

7. Vasina EM, Cauwenberghs S, Feijge MAH, Heemskerk JWM, Weber C, Koenen RR. Microparticles from apoptotic platelets promote resident macrophage differentiation**.** Cell Death Dis. 2011; 2(9)**:**e211-e211.

8. Dieudé M, Bell C, Turgeon J, Beillevaire D, Pomerleau L, Yang B, Hamelin K, Qi S, Pallet N, Béland C, et al. The 20S proteasome core, active within apoptotic exosome-like vesicles, induces autoantibody production and accelerates rejection**.** Sci Transl Med. 2015; 7(318)**:**318ra200-318ra200.

9. Ma Q, Liang M, Wu Y, Luo F, Ma Z, Dong S, Xu J, Dou C. Osteoclast-derived apoptotic bodies couple bone resorption and formation in bone remodeling**.** Bone Res. 2021; 9(1)**:**5-5.

10. Mitra S, Exline M, Habyarimana F, Gavrilin MA, Baker PJ, Masters SL, Wewers MD, Sarkar A. Microparticulate Caspase 1 Regulates Gasdermin D and Pulmonary Vascular Endothelial Cell Injury**.** Am J Resp Cell Mol. 2018; 59(1)**:**56-64.

11. Zheng D, Fan J, Liu X, Dong X, Pan P, Xu L, Zhang X. A Simply Modified Lymphocyte for Systematic Cancer Therapy**.** Adv Mater. 2018; 30(31)**:**1801622.

12. Zhao D, Tao W, Li S, Chen Y, Sun Y, He Z, Sun B, Sun J. Apoptotic body-mediated intercellular delivery for enhanced drug penetration and whole tumor destruction**.** Sci Adv. 2021; 7(16).

13. Zheng L, Hu X, Wu H, Mo L, Xie S, Li J, Peng C, Xu S, Qiu L, Tan W. In Vivo Monocyte/Macrophage-Hitchhiked Intratumoral Accumulation of Nanomedicines for Enhanced Tumor Therapy**.** J Am Chem Soc. 2019; 142(1)**:**382-391.

14. Gupta KH, Goldufsky JW, Wood SJ, Tardi NJ, Moorthy GS, Gilbert DZ, Zayas JP, Hahm E, Altintas MM, Reiser J, Shafikhani SH. Apoptosis and Compensatory Proliferation Signaling Are Coupled by CrkI-Containing Microvesicles**.** Dev Cell. 2017; 41(6)**:**674-684.e5.

15. Wang Y, Pang J, Wang Q, Yan L, Wang L, Xing Z, Wang C, Zhang J, Dong L. Delivering Antisense Oligonucleotides across the Blood‐Brain Barrier by Tumor Cell‐Derived Small Apoptotic Bodies**.** Adv Sci. 2021; 8(13)**:**2004929.

16. Gieseler RK, Marquitan G, Schlattjan M, Sowa JP, Bechmann LP, Timm J, Roggendorf M, Gerken G, Friedman SL, Canbay A. Hepatocyte apoptotic bodies encasing nonstructural HCV proteins amplify hepatic stellate cell activation: implications for chronic hepatitis C**.** J Viral Hepatitis. 2011; 18(11)**:**760-767.

17. Ito F, Kato K, Yanatori I, Murohara T, Toyokuni S. Ferroptosis-dependent extracellular vesicles from macrophage contribute to asbestos-induced mesothelial carcinogenesis through loading ferritin**.** Redox Biol. 2021; 47**:**102174-102174.

18. Ma L, Chen C, Liu D, Huang Z, Li J, Liu H, Kin Kwok RT, Tang B, Sui B, Zhang X, et al. Apoptotic extracellular vesicles are metabolized regulators nurturing the skin and hair**.** Bioactive materials. 2023; 19**:**626-641.

19. Dou G, Tian R, Liu X, Yuan P, Ye Q, Liu J, Liu S, Zhou J, Deng Z, Chen X, et al. Chimeric apoptotic bodies functionalized with natural membrane and modular delivery system for inflammation modulation**.** Sci Adv. 2020; 6(30)**:**eaba2987-eaba2987.

20. Kraynak CA, Yan DJ, Suggs LJ. Modulating inflammatory macrophages with an apoptotic body-inspired nanoparticle**.** Acta Biomater. 2020; 108**:**250-260.

21. Gao Y, Zhang H, Zhou N, Xu P, Wang J, Gao Y, Jin X, Liang X, Lv J, Zhang Y, et al. Methotrexate-loaded tumour-cell-derived microvesicles can relieve biliary obstruction in patients with extrahepatic cholangiocarcinoma**.** Nat Biomed Eng. 2020; 4(7)**:**743-753.

22. Liu Y, Wang J, Zhang J, Marbach S, Xu W, Zhu L. Targeting Tumor-Associated Macrophages by MMP2-Sensitive Apoptotic Body-Mimicking Nanoparticles**.** Acs Appl Mater Inter. 2020; 12(47)**:**52402-52414.

23. Wu Y, Zhang Y, Dai L, Wang Q, Xue L, Su Z, Zhang C. An apoptotic body-biomimic liposome in situ upregulates anti-inflammatory macrophages for stabilization of atherosclerotic plaques**.** J Control Release. 2019; 316**:**236-249.
